# Genetic parenthood and causation: An objection to Douglas and Devolder’s modified direct proportionate genetic descent account

**DOI:** 10.1111/bioe.12639

**Published:** 2019-08-22

**Authors:** César Palacios‐González

**Affiliations:** ^1^ Oxford Uehiro Centre for Practical Ethics University of Oxford UK

**Keywords:** assisted reproduction, cloning, genetic parenthood, Heidi Mertes, in vitro reproduction, mitochondrial replacement therapy, reproduction, stem cell derived gametes

## Abstract

In a recent publication Tom Douglas and Katrien Devolder have proposed a new account of genetic parenthood, building on the work of Heidi Mertes. Douglas and Devolder’s account aims to solve, among other things, the question of who are the genetic parents of an individual created through somatic cell nuclear transfer (i.e. cloning): (a) the nuclear DNA provider or (b) the progenitors of the nuclear DNA provider. Such a question cannot be answered by simply appealing to the folk account of genetic parenthood, according to which the genetic parents of an individual are those individuals who produced the egg and sperm, respectively, which fused to create the embryo. It cannot be so as in cloning there is no fertilization as such. In this article I critically examine Douglas and Devolder’s new account of genetic parenthood and demonstrate that it is vulnerable to counterexamples that exploit the lack of a condition specifying that genetic parents should cause a child’s coming into existence.

## 1 INTRODUCTION

Dolly the sheep is probably the most famous sheep in the world, and she is rightly so as she was the first mammal produced via cloning. In cloning the nucleus of a somatic cell is transferred into an enucleated oocyte (i.e. an oocyte whose nucleus has been previously removed), and then it is activated by external means. If everything goes according to plan the cell will begin to divide, the embryo will be transferred into a womb (under the appropriate biological conditions) and then, after some time, an ‘nearly identical genetic copy’ of the individual who provided the somatic cell (i.e. the nuclear DNA provider) will be born.1Wilmut, I., Beaujean, N., de Sousa, P. A., Dinnyes, A., King, T. J., Paterson, L. A. … Young, L. E. (2002). Somatic cell nuclear transfer. *Nature*, *419*(6907), 583–587. I say a nearly identical genetic copy because whereas the totality of the *nuclear DNA* would come from the nuclear DNA provider, the zygote’s *mitochondrial DNA* would most certainly not come from said nuclear DNA provider, or the provider’s maternal line. This is the case as in mammals mitochondria are matrilineally transmitted.2It is very uncommon for paternal mitochondria to be incorporated into the zygote. Luo, S., Valencia, C. A., Zhang, J., Lee, N. C., Slone, J., Gui, B., … Huang, T. (2018). Biparental inheritance of mitochondrial DNA in humans. *Proceedings of the National Academy of Sciences of the United States of America*. *115*(51), 13039–13044; Schwartz, M., & Vissing, J. (2002). Paternal inheritance of mitochondrial DNA. *New England Journal of Medicine*, *347*(8), 576–580. For the clone to be an identical genetic copy, *sensu stricto*, of the nuclear DNA provider the latter, or someone from their maternal line, should also provide the would‐be enucleated egg that in turn contains the mitochondria.

After the birth of Dolly scientists around the world started to try to clone other mammals, and up to this date cats, coyotes, camels, dogs, goats, horses, mice, monkeys, pigs, rabbits and rats have already been cloned. Furthermore, one consequence of Dolly’s birth was that people got even more interested in the question of whether human beings *could* be cloned, and if so *whether it would be morally permissible to do so*.3Devolder, K. (2017). Cloning. In Zalta, E. N. (Ed.). *The Stanford encyclopedia of philosophy*. Stanford, CA: Stanford University. https://plato.stanford.edu/entries/cloning/
 Broadly speaking, two positions emerged. On the one had there are those who argue that it would be immoral to clone human beings, and on the other hand there are those who argue that under certain circumstances it would be morally permissible to do so. An example of the former is Leon Kass: ‘Mass‐scale cloning of the same individual makes the point vividly; but the violation of human equality, freedom, and dignity is present even in a single planned clone’.4Kass, L. R. (1998). The wisdom of repugnance: Why we should ban the cloning of humans. *Valparaiso University Law Review, 32*(2), 697. An example of the latter is Carson Strong, who argued that in certain circumstances it would be ethical for infertile couples to resort to cloning, as a way of having kin that were genetically related to one of them.5Strong, C. (1998). Cloning and infertility. *Cambridge Quarterly of Healthcare Ethics*, *7*(3), 279–293.


One issue that becomes patent when we reflect on the possibly of employing human cloning for ‘reproductive purposes’ is that cloning *does not fit* our folk conception of genetic parenthood. According to this folk conception, the person who provided the egg and the person who provided the sperm, which fused and created an embryo, are the genetic parents of the created embryo. Cloning is problematic because in it no sperm and egg fuse, and thus the question of who are the genetic parents of any given clone arise: (a) *the person* who provided the nuclear DNA, or (b) *the progenitors* of the person who provided the nuclear DNA. Interestingly, Leon Kass noticed that reproductive cloning complicated our traditional understanding of human reproduction: ‘Asexual reproduction, which produces “single‐parent” offspring, is a radical departure from the natural human way, confounding all normal understandings of father, mother, sibling, grandparent, etc., and all moral relations tied thereto.’6Kass, op. cit., note 4, p. 690.


In a recent publication Tom Douglas and Katrien Devolder have proposed a new account of genetic parenthood. Their account, which builds on the work of Heidi Mertes,7Mertes, H. (2014). Gamete derivation from stem cells: Revisiting the concept of genetic parenthood. *Journal of Medical Ethics*, *40*(11), 744–447. aims to solve the question of who are the genetic parents of an individual created through cloning. They defend the view that the progenitors of the nuclear DNA provider are the clone’s genetic parents. In this article I critically examine Douglas and Devolder’s new account of genetic parenthood. Firstly, I show that Douglas and Devolder draw an incorrect conclusion when they apply their new account of genetic parenthood to answer the question of whether an egg donor is a genetic parent, in cases where the egg used in the reproductive procedure has been enucleated (e.g. maternal spindle transfer, cloning). Secondly, I demonstrate their account is vulnerable to counterexamples that exploit the absence of a condition specifying that genetic parents should cause the child’s coming into existence.

The article proceeds as follows. In the second section I briefly present and explain why previous accounts of genetic parenthood are flawed. In the third section I present Douglas and Devolder’s new account of genetic parenthood and show that they draw the wrong conclusion when faced with cases where an egg has been enucleated for a reproductive purpose. In the fourth, and final, section I present a case that shows that their account of genetic parenthood is found wanting.

## 2 ACCOUNTS OF GENETIC REPRODUCTION

In this section I review the accounts of genetic parenthood that Heidi Mertes investigated in her article ‘Gamete Derivation from Stem Cells: Revisiting the Concept of Genetic Parenthood’; and explain why they are found wanting. I begin with Mertes, as Douglas and Devolder do likewise. The first account that she presents can be named the informational account of genetic parenthood:

[A] child is my genetic child when it has 50% of my DNA or when it has 23 of my chromosomes. This 50% overlap of genetic material is, for example, what is looked into when performing a paternity or maternity test.8Ibid, p. 744.

There are several problems with this account, as it focuses on information overlap. Foremost, under it my siblings would be my genetic parents and vice versa. This would be the case as I share 50% of the information contained in my genetic nuclear material with my siblings. Furthermore, it can also be the case that I share 50% of my nuclear DNA, in the informational sense, with someone who is not a close relative of mine, and that would entail that she is my genetic child or parent.

A more promising account, which Mertes discusses and which was first presented by Avery Kolers,9Kolers, A. (2003). Cloning and genetic parenthood. *Cambridge Quarterly of Healthcare Ethics*, *12*(4), 401–410. can be named the Direct Derivation Account of genetic parenthood: ‘X is a genetic child of Y if X is directly derived from Y’s genes’.10Mertes, op. cit., note 7, p. 744.


The direct derivation condition rules out the possibility of my siblings being my genetic parents, as I was not directly derived from them, and vice versa. As Kolers asserts: ‘[d]erivation is fundamentally a *causal* relationship; the offspring is as it is *because* of its relationship to its parents, whereas the inverse is not true’.11Kolers, op. cit., note 9, p. 402. Mertes contends that the problem with this account is that it leads to counterintuitive conclusions when we examine two reproductive cloning cases.12In what follows I will bracket the question whether the egg donor, for the cloning procedure, is a genetic parent under the Direct Derivation Account. I do so as I will expand on this issue later on. Let us, following Douglas and Devolder, call the first case the Cloned Child:

[A] couple (Mr and Mrs X) may become infertile after already having conceived one genetically related child (Y). As they long for a second child, they opt to clone their existing child Y, which results in the birth of Z.13Mertes, op. cit., note 7, p. 745.

According to the Direct Derivation Account, Mr and Mrs X *are not* the genetic parents of Z. They are not so as Z was not *directly derived* from their genes. On the other hand, Y *is* the genetic parent of Z, as Z was directly derived from Y’s genes. Nevertheless, according to Mertes, in this scenario ‘it is most likely that Mr and Mrs X will “feel” like the genetic parents of Z, whereas Y is unlikely to think of herself as Z’s mother.’ Let us now consider the second case that Mertes presents, that I will call Cloned Parent, also following Douglas and Devolder: ‘Mr and Mrs X decide to clone Mr X instead of Y, resulting in child Q.’14Ibid.


According to the Direct Derivation Account, Mr X is the *only* genetic parent of Q. He is so as Q’s genes were directly derived only from Mr X. Now, Mertes notices that in this case *the nuclear DNA provider and the clone*

do not share 50% of DNA, but 100%. The people who would pass [in Cloned Parent] a maternity/paternity test would be the genetic parents of Mr X. In this second scenario, both Mr X and his parents might consider themselves as Q’s genetic parents and both would have good arguments (either a contribution of 50% DNA or direct derivation) to support their claim.15Ibid.

The overall issue here, according to Mertes, is that we have *contradictory intuitions* on similar cloning scenarios. On the one hand, in Cloned Child we seem to support the idea that *the progenitors of the nuclear DNA provider are the genetic parents of the clone*. But on the other hand, in Cloned Parent we seem to support the idea that *the nuclear DNA provider is the genetic parent of the clone*. Of course, holding both statements as true at the same time is contradictory.

After presenting these two cloning cases Mertes does not bite the bullet and accept that, in Cloned Child, Z’s genetic parent is Y. And she also does not try to revise the Direct Derivation Account in order to solve this purported contradiction in our intuitions. What she does is assert that these two cases show that ‘[t]here is no fixed, scientific, everlasting criterion of genetic parenthood that everyone can agree upon’ and that ‘the term genetic parenthood is not value‐free, but dependent on personal intuitions, intentions or judgements’.16Ibid. Let us now move to Douglas and Devolder’s account of genetic parenthood.

## 3 THE MODIFIED DIRECT PROPORTIONATE GENETIC DESCENT ACCOUNT

Douglas and Devolder contend that Mertes’s conclusion about genetic parenthood is premature, in the sense that she has not shown that genetic parenthood is ‘a subjective concept that depends on the views of people about what sorts of genetic relation matter’.17Douglas, T., & Devolder, K. (2018). A conception of genetic parenthood. *Bioethics*, *33*(1), 54–59, p. 4. Rather than falling for this subjectivist account of genetic parenthood they propose a new account, one that can deal with the Cloned Child and the Cloned Parent cases. But importantly, their account is intended to be one ‘that captures the concept of genetic parenthood implicit in everyday usage’.18Ibid, p. 1. Before engaging with Douglas and Devolder’s account, it is important to notice that they do not start from Mertes’s presentation of the Direct Derivation Account, but rather from a *revised* version of it: ‘Direct Genetic Descent: P is C’s genetic parent if and only if (a) C’s genes derived from P’s genes, and (b) not through deriving from the genes of some third, intervening individual, M.’19Ibid, p. 3.


They start from this revised version in order to avoid a possible counterexample:

Suppose a sperm from P1 is used to fertilize an egg from P2. The resulting zygote then has its DNA removed and replaced by DNA from some other individual T. This zygote is then carried to term and eventually a child, C, is born. There is a sense in which C derives directly from P1 and P2’s genes; those genes governed the development of gametes, which created a zygote from which C developed. But C’s genes do not derive from P1 and P2’s genes, they derive instead from T’s genes, and this surely prevents P1 and P2 from qualifying as C’s genetic parents.20Ibid.

Douglas and Devolder’s Direct Genetic Descent, which is a revised version of Mertes’s presentation of the Direct Derivation Account, runs into the same issues mentioned above when we consider the Cloned Parent and Cloned Child cases. Now, Douglas and Devolder offer a refined version of Direct Genetic Descent, which seems to answer the question of who are the clone’s genetic parents, while at the same time maintaining the concept of genetic parenthood implicit in everyday usage:

Direct Gametic Genetic Descent: P is C’s genetic parent if and only if (a) C’s genes derived from P’s genes, (b) through a gamete produced by P, and (c) not through deriving from the genes of some third, intervening individual, M.21Ibid, p. 4.

Douglas and Devolder here introduce the condition that the genetic derivation must be via a gamete produced by the intending genetic parent. Thus it follows from this account that the nuclear DNA provider, in cloning cases where the enucleated egg does not come from said nuclear DNA provider, *is not a genetic parent* of the clone. In such cases he or she is not a genetic parent as the clone was not derived from one of their gametes. In Cloned Child, Y, who is the nuclear DNA provider, is not the genetic parent of clone Z. And in Cloned Parent, Mr X, who is the nuclear DNA provider, is not the genetic parent of Q.

Douglas and Devolder also conclude, following the Direct Gametic Genetic Descent account, that in cases where we employ a mitochondrial replacement technique the egg donor is not a genetic parent of the created child. Direct Gametic Genetic Descent ‘also allows us to evade the conclusion that P3 (the mitochondrial donor) is the genetic parent of C4 (the mitochondrial recipient) in Mitochondrial Donation.’22Ibid. Let us remember that, broadly speaking, mitochondrial replacement techniques are those where the nuclear DNA of the intending mother (or intending parents) is transferred from an egg (or zygote) with deleterious mitochondrial DNA mutations, to an enucleated egg (or zygote) that possesses healthy mitochondria.23Palacios‐González, C. (2017). Ethics of mitochondrial replacement techniques: A Habermasian perspective. *Bioethics. 31*(1), 27–36. It is also relevant to bear in mind that mitochondrial DNA corresponds to, roughly, 0.1% of the whole DNA content of the human organism.

Douglas and Devolder are mistaken in their application of Direct Gametic Genetic Descent to mitochondrial replacement techniques cases. In such an account the egg donor for a mitochondrial replacement technique *is a genetic parent*. Why? Because the child’s mitochondrial genes were derived from the egg donor’s genes (Douglas and Devolder does not specify if there is a minimum of genes that must be transmitted for someone to classify as a genetic parent), from a gamete produced by the egg donor, and not by deriving such genes from the mitochondrial genes of some third, intervening individual, M. And the same rationale would apply to the egg donor in cases of reproductive cloning: she would be a genetic parent of the clone, regardless of the fact if she is also the nuclear DNA provider.

Now, Douglas and Devolder assert that from Direct Gametic Genetic Descent it follows that neither of the clones (i.e. Q and Z) has genetic parents: Direct Gametic Genetic Descent ‘implies that the clones created in Cloned Child and Cloned Parent lack any genetic parents, since their genomes were not inherited via gametes’.24Douglas & Devolder, op. cit., note 17, p. 5.
*Stricto sensu* Douglas and Devolder are incorrect. The clones created in Cloned Child and Cloned Parent have at least one genetic parent: the egg donor.

Bracketing the previous issue regarding egg donors, Douglas and Devolder assert that Direct Gametic Genetic Descent is not only problematic because the clones would not have genetic parents, which they do. They contend that Direct Gametic Genetic Descent is flawed because there are other cases that are ‘closer to those of normal human reproduction’ that also create problems for such an account. As a point in case, imagine that the following is possible: we enucleate a zygote, and we replace its nuclear material with the genetic material obtained from two somatic cells taken from individuals A and B. In this case each individual contributes 50% of the nuclear DNA material.25Ibid. If this were ever to happen then, under direct gametic genetic descent, we would have to accept that any resulting child from this biotechnology, let us call it (following Douglas and Devolder) Two‐Donor Genome Transplantation, would not have genetic parents. Yet it seems that in this case A and B would be the genetic parents.

In order to solve the problem posed by Two‐Donor Genome Transplantation, Douglas and Devolder propose to replace the condition of genetic inheritance via gametes with a condition of genetic inheritance of some determined proportion from parent to child. They call their new account Direct Proportionate Genetic Descent:

P is C’s genetic parent if and only if (a) some proportion X of C’s genes derived from P’s genes and (b) not through deriving from the genes of some third, intervening individual, M.26Ibid.

If proportion X, which they do not specify, were to be <100% of the *nuclear DNA*, then under this account the nuclear DNA provider is not the clone’s genetic parent. And if proportion X were to be > 0.1% *of the whole DNA* then the egg donor, in cases where we employ maternal spindle transfer, for example, would not be a genetic parent. Now, the upshot of the Direct Proportionate Genetic Descent account is that it does not entail the absurd conclusion that in Two‐Donor Genome Transplantation the resulting child has no genetic parents. According to this account A and B would be the child’s genetic parents. This would be the case if the proportion of genetic material established by this account (i.e. X) is set to include the proportion that obtains in cases of sexual reproduction.

Even though Direct Proportionate Genetic Descent can accommodate certain counterexamples, Douglas and Devolder maintain that according to it the clones created in Cloned Parent and Cloned Child still do not have genetic parents:

However, Direct Proportionate Genetic Descent remains too stringent in the cases of Cloned Child and Cloned Parent, for it continues to imply that the clones produced in these cases have no genetic parents – an implication that Mertes and Sparrow find implausible.27Ibid.

It is important to note that this conclusion *does not necessarily* follow from their account. It does not do so as proportion X could be a proportion of genetic material in the range between 50% and 100%. The fact that the nuclear DNA provider passes ‘too much’ of her genetic material does not rule her out from being a genetic parent *in principle*. Regardless of how we solve the previous point, under Direct Proportionate Genetic Descent the *genetic parents* of the nuclear DNA provider (i.e. the parents of Mr X and Y) cannot be regarded as the genetic parents of the clone (i.e. Q and Z); as in such cases they – the genetic parents of the nuclear DNA provider – are the ‘*third intervening individual’*.

Douglas and Devolder contend that it is possible to revisit the conditions established in Direct Proportionate Genetic Descent and soften them, so we arrive at an account of genetic parenthood that does not conclude that in Cloned Parent and Cloned Child the clones *do not have genetic parents*. In the previous paragraph I have shown that this ‘softening’ is not necessary, but adopting such solution would entail abandoning the concept of human genetic parenthood implicit in everyday usage. The way in which Douglas and Devolder weaken Direct Proportionate Genetic Descent is by *abandoning* the idea that there cannot be a third intervening individual. Now they accept that *in some instances* there can be a third individual involved in the reproductive endeavour. But, interestingly, this third individual *is not a genetic parent*, but what can be described as a *bridge* between progenitors and descendants. For example, in Cloned Child case they ‘propose that the intervening individual (C1) *does not break the genetic parenthood relation* [between P1 and P2, and C2] in this case because she passes on too much of her genetic information to C2 [emphasis added]’.28Ibid. Douglas and Devolder formalize their revised account as follows:

*Modified Direct Proportionate Genetic Descent*: P is C’s genetic parent if and only if (a) *some proportion* X of C’s genes derived from P’s genes and (b) not through deriving from the genes of some third, intervening individual M *from whom C derived proportion Y of his genes*.29Ibid.

Douglas and Devolder accept that ‘the *proportion* of genes’ will most probably not be a set figure but rather a range, and that this range could have fuzzy boundaries. Before I show why this account of genetic parenthood is found wanting, it is relevant to mention that according to it egg donors, both for cloning and mitochondrial replacement procedures, are not *in principle* ruled out as genetic parents. Why? Because, as stated above, the authors do not specify the proportion of genes that must be transmitted for someone to classify as a genetic parent. If the proportion were ≥ 0.1% of the whole DNA content of the human organism then the egg donor would be the genetic parent of the resulting child. This would be so as the mitochondrial genes of any resulting child would have been derived from the egg donor’s genes, and not at all through some third intervening individual M.30I thank an anonymous reviewer for bringing this point to my attention.


## 4 THE CASE AGAINST THE MODIFIED DIRECT PROPORTIONATE GENETIC DESCENT ACCOUNT

Even if Douglas and Devolder’s account avoids what they consider to be a counterintuitive conclusion (i.e. that Mr X is Q’s genetic parent, and that Y is Z’s genetic parent), they run into a more severe problem for not having a *causation condition* built into their account. In order to appreciate this issue consider the following case, which I will call ‘Genome Editing ’. Alfred and Betty want to have a child. They have been unsuccessful in achieving their goal through sexual intercourse, as both of Betty’s fallopian tubes are completely blocked. Alfred and Betty decide to resort to Charles, a fertility expert. Charles prescribes Betty some fertility drugs and then proceeds to surgically retrieve her eggs. Once the eggs have been retrieved he uses Alfred’s sperm in order to carry out in vitro fertilization, and zygote E is produced. After the IVF procedure Charles does not transfer the zygote back to Betty, but rather he lets it grow in his lab for 3 days. On the fourth day Charles uses a Genome Editing technique in order to modify E’s cells. In this case he inserts 10% of his own nuclear genes into E’s cells. Finally, Charles transfers the genetically modified embryo to Betty, and after some months E is born.

According to Modified Direct Proportionate Genetic Descent both Alfred and Betty are E’s genetic parents, if ‘proportion X’ is set to include a range between 5% and 50% of the total nuclear material. They are so in that 45% of E’s nuclear genes were derived from each one of them, and not through deriving from the genes of some third intervening individual. The question to answer now is whether Charle, the fertility doctor, is E’s genetic parent. When E is first created Charles *is not* his genetic parent, as he does not satisfy condition (a). But, Charles *becomes E’s genetic parent* per means of a Genome Editing technology when E is 4 days old*.* This is the case as 10% of E’s genes were derived from Charles’ genes, and not through deriving them from the genes of some third intervening individual.

The fact that Charles *becomes* E’s genetic parent shows that there is something wrong with Modified Direct Proportionate Genetic Descent. The problem is that this account allows for an adult *to become* the genetic parent of an *already existing individual*; and this cuts against one of the necessary conditions of genetic parenthood: that one is one of the material causes of an individual coming into existence. Adopting a stance that maintains that such a causation condition is not necessary entails a radical revisionism of the concept of genetic parenthood, a revisionist position that needs to be defended.

At this point someone could present two objections to my Genome Editing case. First, they could argue that given that early embryos can twin they are not individuals, in the sense of being unified biological organisms, and thus Genome Editing does not show that such an account is found wanting.31Devolder, K., & Harris, J. (2007). The ambiguity of the embryo: Ethical inconsistency in the human embryonic stem cell debate. *Metaphilosophy*, *38*(2–3), 153–169. There are three ways in which I can address this objection. Firstly, I could show that early embryos are in fact individuals, but I do not have enough space to defend such a view (see Mathew Liao (2010) for a defence of such position).32Liao, S. M. (2010). Twinning, inorganic replacement, and the organism view. *Ratio*, *23*(1), 59–72. Secondly, my case could just be recast as referring to embryos that have passed the point where twining is possible. Third, Genome Editing works in non‐embryonic cases as well. Imagine that rather than being a 3‐day old embryo, E is a 1‐year old baby. If Charles were to edit his genome, at that point, so that now 10% of E’s genome derived from Charles then Charles, again, would become E’s genetic parent, but this is absurd.

The second objection holds that in Genome Editing it is not the case that Charles *becomes* the genetic parent of an *already existing individual*, this is not so because replacing 10% of E’s genes would destroy E and create a new individual F.33I thank an anonymous reviewer for raising this objection. In other words, E’s numerical identity would not survive the extent of such procedure. If this were so then Charles would be the genetic parent *of the recently created individual F*, in as much as 10% of F’s genes derived from Charles’ genes and not through deriving them from some third intervening individual. According to this objection Genome Editing does not even get off the ground.

In order to respond to this objection the first thing I need to do is provide an account of what an organism is. According to Liao, a being X is essentially an organism if

a) X begins to exist when the capacity to regulate and coordinate metabolic and other life processes is there; b) X persists as long as there is what may be called ‘organismic continuity,’ which is the continuing ability to regulate and coordinate metabolic and other life processes; and c) X ceases to exist when the capacity to regulate and coordinate metabolic and other life processes is permanently gone.34Liao, S. M. (2006). The organism view defended. *The Monist*, *89*(3), 334–350.

Now, it could be the case that all our DNA is necessarily required for the capacity to regulate and coordinate metabolic and life processes to be there; and thus, if some of it were to be replaced by similar DNA, but from a different origin, then the original capacity to regulate and coordinate would be destroyed and a new capacity would be created. Even when it is intuitively appealing the former is incorrect. According to recent research 75% of our DNA is non‐coding DNA.35Graur, D. (2017). An upper limit on the functional fraction of the human genome. *Genome Biology and Evolution*, *9*(7), 1880–1885. This means that Charles could edit 10% of E’s non‐coding DNA, and this would not affect E’s capacity to regulate and coordinate metabolic and life processes thus, also, not affecting E’s numerical identity. Furthermore, my response to this second objection holds true even if it were to be the case that the findings of the Encyclopedia of DNA Elements project, which were able to assign biochemical functions to 80% of the genome, were true.36The ENCODE Project Consortium. (2012). An integrated encyclopedia of DNA elements in the human genome. *Nature*, *489*(7414), 57–74. Let me finish by saying that Genome Editing shows that Douglas and Devolder’s Modified Direct Proportionate Genetic Descent account of genetic parenthood is presently found wanting, and that they need to do more work in order for it to be a plausible account of genetic parenthood.

